# Editorial: The global phenotypic diversity of HIV-1: implications for pathogenesis, vaccine, and cure

**DOI:** 10.3389/fimmu.2025.1572732

**Published:** 2025-02-28

**Authors:** Azam Bolhassani

**Affiliations:** Department of Hepatitis, AIDS and Blood-borne Diseases, Pasteur Institute of Iran, Tehran, Iran

**Keywords:** HIV-1, phenotypic diversity, pathogenesis, vaccine, antiretroviral therapy, drug resistance

Antiretroviral therapy (ART) is under continuous development with the goal of long-term control of HIV replication using effective and well-tolerated drugs. On 29 February 2024, the WHO published an updated report about HIV drug resistance.

Despite the clinical success of antiretroviral therapy, there is no cure for HIV infection, yet. As reported, HIV vaccines in development are in early-stage clinical trials (1). Indeed, the global and regional genetic diversity of HIV-1 (*i.e.*, its high genetic mutation and recombination rates) are a main challenge for HIV vaccine development. Thus, investigating the distribution of HIV subtypes/clades in different regions is essential.

To date, several immunotherapy approaches were considered to activate the immune system (*e.g.*, CD8^+^ T cells and NK cells) and eradicate latently HIV-infected cells (*i.e.*, elimination of the HIV reservoir) including: a) reactivation of the latently HIV-infected cells using epigenetic modulators (2, 3) or immunostimulators (*e.g.*, toll-like receptor (TLR) agonists) (4, 5); b) combination of a potent latency reversal agent (*e.g.*, Romidepsin) with an immune modulator (*e.g.*, a therapeutic vaccine) (6 and 7); and c) the use of chimeric antigen receptors (CARs) for directly killing HIV-infected cells (8, 9). Thus, it is critical to determine which immunotherapy approaches have an important effect in elimination of latently HIV-infected cells.

At present, some studies aim to develop preventive HIV vaccines and also overcome antiretroviral drug resistance as a cure for HIV infection. Current approaches in the management of HIV infection include prevention/reversion of viral latency, modulation of immune responses, development of novel vaccines, and improvement of antiretroviral drugs in HIV patients (10, 11).

To address these critical areas in HIV management, this Research Topic focused on: a) Phenotypic and pathogenicity properties of newly identified/emerged HIV-1 strains; b) Susceptibility to innate/adaptive immune responses and broadly neutralizing antibodies (bNAbs); c) Treatment outcomes and drug resistance; and d) Outcomes of clinical trials, including vaccine, treatment, and functional cure. In total, four articles were published in association with these topics as follows. In Paper 1, Pei et al. recommended resistance testing before ART to improve effective treatment and reduce the spread of resistant viruses. Moreover, molecular networks were highlighted for their utility in identifying transmission clusters and enabling more precise interventions. In this study, authors detected 10 HIV-1 subtypes in heterosexually transmitted patients, and 7 subtypes in homosexually transmitted patients. CRF07_BC and CRF01_AE were the most common subtypes among patients. The drug resistance rates of heterosexual and homosexual individuals were 45.34% and 33.33%, respectively. The molecular transmission network showed that the clustering rates of homosexual and heterosexual individuals were 52.78% and 39.13%, respectively. In paper 2, Ma et al. showed that longitudinal trajectory analysis of multiple immune indicators (*i.e.*, CD4 count, CD8 count and CD4/CD8 ratio) can be used to guide targeted interventions among susceptible populations. They described that the combination of CD4 count, CD8 count and CD4/CD8 ratio is essential to predict risk of mortality among HIV-positive individuals up to 96 months following initiation of ART. Among the four study groups (group 1: low CD4 and CD4/CD8 inversion, group 2: high CD8 and CD4/CD8 inversion, group 3: slow recovery of CD4 and CD4/CD8 inversion, and group 4: rapid increase of CD4 and normal CD4/CD8), groups 1 and 3 had increased risk of mortality. In addition, immune recovery was slower in male than in female and in older individuals than in younger individuals.

In paper 3, Dirajlal-Fargo et al. indicated that children with perinatally acquired HIV (PHIV) had higher T cell activation and damage in the gut integrity despite ART and viral suppression more than two years. Therefore, authors highlighted extensive research on development of newer ART regimens and early ART initiation in PHIV to decrease inflammation and prevent long-term comorbidities. In addition, it is critical that the links between intestinal barrier function, intestinal microbiota composition, and immune activation are determined to decrease potential comorbidities in HIV-infected children. In paper 4, Pan et al. represented the first molecular transmission network analysis of the HIV-1 subtype B circulating in China. They highlighted the importance of the combined molecular surveillance and epidemiology. Their data indicated that more than 99% of subtype B sequences belonged to Thai B. Phylogenetic and molecular network analyses revealed a common origin with neighboring regions in mainland China. In general, at least two coexisting transmission routes in most transmission clusters involve a greater challenge in controlling the prevalence of HIV-1 infection.

Altogether, the complexity of HIV-1 diversity establishes increasing challenges for development of preventive, therapeutic and functional cure approaches towards the goal to end the pandemic. We hope that four published scientific articles could provide new outlooks on studying HIV and AIDS from different perspectives including resistance testing before ART, longitudinal trajectory analysis of multiple immune indicators, development of newer ART regimens and early ART initiation in PHIV, and the importance of combined molecular surveillance and epidemiology.
